# The effect of different calcium silicate-based pulp capping materials on tooth discoloration: an in vitro study

**DOI:** 10.1186/s12903-021-01677-y

**Published:** 2021-07-02

**Authors:** Ahmad S. Al-Hiyasat, Dana M. Ahmad, Yousef S. Khader

**Affiliations:** 1 Department of Conservative Dentistry, Faculty of Dentistry, Jordan University of Science and Technology, P.O. Box 3030, Irbid, 22110 Jordan; 2 Department of Public Health and Community Medicine, Faculty of Medicine, Jordan University of Science and Technology, Irbid, Jordan

**Keywords:** Calcium silicate materials, Pulp capping, Tooth discoloration

## Abstract

**Background:**

Variation in the composition of calcium silicate-based pulp capping materials could influence the discoloration potential of some of these materials, thus affecting the color and aesthetic appearance of the coronal tooth structure. Furthermore, contact with blood if hemostasis is not fully achieved may enhance this discoloration for some materials. Therefore the aim of this study was to evaluate in vitro the color change of coronal tooth structure after placing various calcium silicate-based materials in the pulp chamber in the presence or absence of blood.

**Mehtods:**

Maxillary extracted premolars (n = 144) were sectioned and the crowns were separated from the roots. Pulp chambers were prepared to a standard size and then the tested materials (GMTA Angelus, ProRoot WMTA, Biodentine, TheraCal, and TotalFill) were placed with saline or with blood. Color change was assessed by spectrophotometry; prior to and after material placement at different time intervals of 24 h, 1 week, 1 month, 3 months, and 6 months. Color change (ΔE) values were calculated and statistically analyzed.

**Results:**

In the saline groups, Biodentine caused the least color change, while GMTA and WMTA caused the highest color change which were significantly different from the others (*p* < 0.001), TotalFill and TheraCal caused moderate changes. Adding blood increased the ΔE overall the tested materials to various degrees. Biodentine was the most affected by the blood, while MTA groups were the least affected, followed by TotalFill and then TheraCal. The increase in ΔE was significant over time up to 3 months, after which the increase was not significant.

**Conclusions:**

Overall, WMTA and GMTA caused the most severe discoloration. In saline, Biodentine caused the least discoloration, but it was the most affected by the presence of blood, although it still caused the least discoloration similar to that observed with TotatFill. TheraCal caused moderate discoloration but more than that caused by Biodentine and TotalFill.

## Background

Vital pulp therapy has entered a golden era by the invention of mineral trioxide aggregate (MTA), which is based on Portland cement, whose components are tricalcium silicate, dicalcium silicate and tricalcium aluminate [1]. MTA was considered to be the material of choice in cases of vital pulp therapy because of its favorable properties such as it’s biocompatibility with minimal cytotoxicity, bioinductivity with hard tissue formation barrier, superior sealing ability, and few tunnel defects in dentinal bridge formation [1–4]. However, one of the drawbacks of MTA is its discoloration potential. It has been reported that the presence of bismuth oxide in the MTA composition, which is added as a radiopacifier is responsible for this discoloration [5, 6]. Other metal oxides in the composition of MTA have also been implicated as the source of discoloration, such as iron oxide, aluminum oxide, and magnesium oxides [7]. In addition, blood contamination that may occur in vital pulp therapy procedures could be considered as an additional causative factor for discoloration [8–10].

Recently, new calcium silicate-based materials have been introduced into the endodontic market, and possess comparable biological and mechanical properties to MTA, such as Biodentine (Septodont, Saint Maur des Fosses, France),TheraCal LC (Bisco Inc, Schamburg, IL, USA) and premixed bioceramics such as TotalFill (FKG, La-Chaux-de-Fonds, Switzerland). The manufacturers of these products are claiming that their products have the ability to overcome the drawbacks of MTA including tooth discoloration potential [11–13]. Thus, the discoloration effect of the various calcium silicate-based materials was of interest to the dental clinicians and researchers, and many of the review studies have addressed this topic concluding that; some of the these materials have a high potential of tooth discoloration, while some have much less discoloration effect, and this variation was related to the differences in the compositions of these materials, therefore, they recommended more laboratory and clinical research studies in this field [14–16].

The composition of Biodentine mainly consists of tricalcium silicate, dicalcium silicate, calcium carbonate, and zirconium oxide; and the liquid consists of calcium chloride which is a setting accelerator and water-reducing agent in aqueous solution [12].

TheraCal is a light-cured, resin-modified calcium silicate-based material. It consists of tricalcium silicate particles in a hydrophilic monomer that stimulates hydroxyapatite and secondary dentin bridge formation, through calcium release and an alkaline pH. The composition also contains barium zirconate as a radiopaque component [13, 17].

TotalFill is a premixed bioceramic material consisting of calcium silicate, zirconium oxide, tantalum oxide, calcium phosphate monobasic, and filler agents. The material comes in different formulated consistencies; syringable paste, condensable putty, and fast set putty [18, 19].

The bismuth oxide in MTA has been replaced by other constituents in the aforementioned materials, such as zirconium oxide, barium zirconate, and tantalum oxide, which have been incorporated into the formulations of these materials, serving as radioopacifier. So the question is, could these variations in the composition of these materials reduce the potential discoloration effect that is associated with MTA, and whether these additions would be affected by the presence of blood, in case of contamination during the clinical procedure of vital pulp therapy, if complete hemostasis is not achieved. Therefore, the aim of the present study was to evaluate -*in vitro-* the color change of coronal tooth structure at different time intervals after placing various calcium silicate-based pulp capping materials into the pulp chamber space in the presence or absence of blood. We hypothesize that the different compositions of calcium silicate materials may cause tooth discoloration to various degrees over time, and contamination with blood will increase the discoloration effect of these materials.

## Methods

### Samples preparation

One hundred forty four human maxillary premolars extracted for orthodontic reason from comparable age group (16–24 years) of patients, who attended the Faculty of dentistry orthodontic clinics at Jordan University of science and technology, were collected and stored in distilled water. The teeth were fully formed and inspected under a microscope (Carl Zeiss Surgical, Oberkochen, Germany) to ensure the absence of cracks, fractures, caries, and/or any defects. Teeth were cleansed and polished with pumice, then sectioned horizontally at 1 mm apical to the cemento-enamel junction, using a diamond disk on straight hand piece. The pulp chamber of the crown was chemo-mechanically cleaned and prepared using a high speed cylindrical diamond bur (Teka Dental Technology, Milano, Italy) with water spray. The cavity was then standardized to have a depth of 5 mm and leaving 3 mm of buccal thickness, followed by irrigation with 2.5% NaOCl (5 ml) and a final rinse with normal saline (5 ml).

Teeth were randomly assigned into two main groups of 72 teeth in each group; one group was used to test the materials with saline, while the other group was used to test the materials with blood. The samples in each group were then randomly divided into six groups (n = 12), five groups for the materials tested and one as a control. The groups were labeled according to the materials that were tested namley: GMTA (Angelus Solucoes Odontologicas, Londrina, Brazil), ProRoot WMTA (Dentsply Tulsa Dental, Johnson City, USA), Biodentine (Septodont, Saint Maur des Fosses, France), TheraCal LC (Bisco Inc, Schamburg, IL, USA), and TotalFill BC RRM putty (FKG, La-Chaux-de-Fonds, Switzerland), and the control groups with none of the tested materials.

**Fig. 1:**
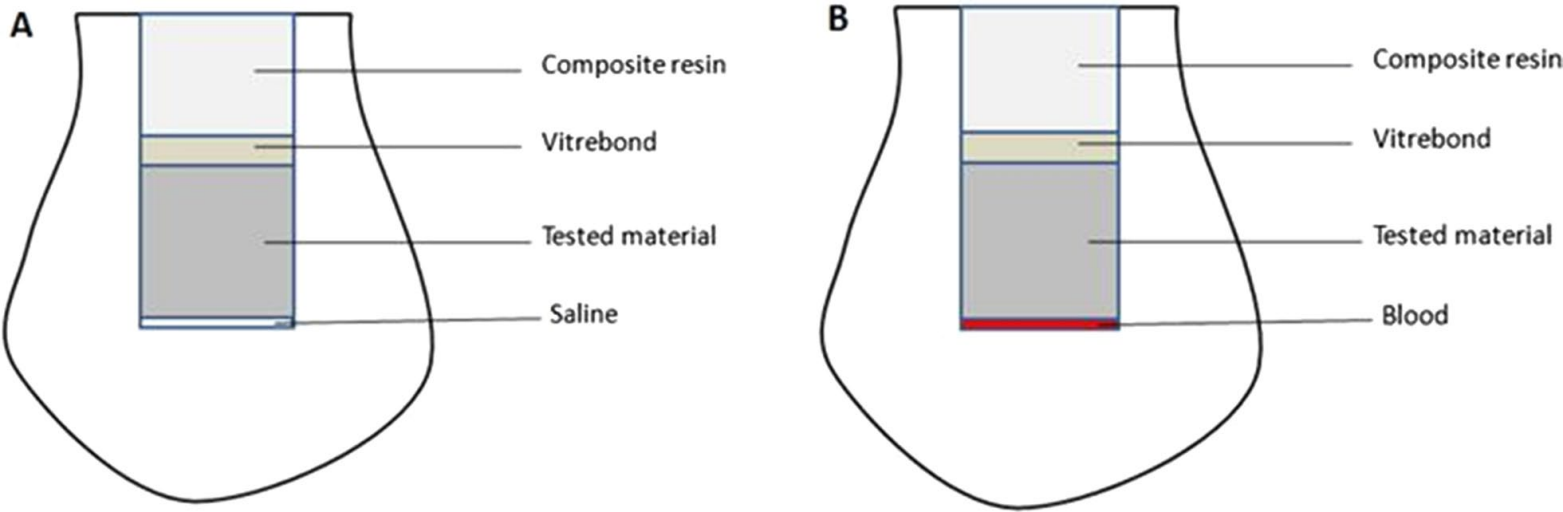
Illustration for the experimental setup: **A** Saline groups; **B** Blood groups

Before material placement, 1.5 μL of either saline or blood was placed into the cavity. The blood was obtained from one of the authors (DMA) who performed the laboratory work and stored in sterile tubes (Vacuette®; Greiner Bio-One, Kremsmünster, Austria), spray-coated with the anticoagulant K3EDTA to prevent clotting. Materials were then prepared according to the manufacturers recommendation and placed into the pulp chamber cavity with a thickness of 3-mm (except the TheraCal group). Then a thin layer of Vitrebond (3M ESPE, 3M Deutschland Gmbh, Neuss-Germany) was applied, followed by 2 mm layer of light cured composite resin shade A3 (3M ESPE, 3M Deutschland Gmbh, Neuss-Germany) (Fig. 1).

TheraCal LC was applied in 3 layers of 1 mm in thickness, each layer was light-cured for 20 seconds, a thin layer of Vitrebond was applied followed by a 2 mm layer of composite resin as the other groups. Negative and positive control specimens were filled with cotton pellet moistened with normal saline (12 μL) or blood (12 μL) respectively, and 2mm thickness layer of Vitremer shade A3 (3M ESPE, 3M Deutschland Gmbh, Neuss-Germany) was applied over the cotton pellet to fill and seal the cavity.

Each tooth was then stored separately in a labelled plastic cuvette in an incubator at 37 °C in 100% humidity in distilled water throughout the study.

### Color assessment

A spectrophotometer (VITA Easyshade compact; VITA Zahnfabrik, Bad Säckingen, Germany) was used for color measurements. To ensure the reproducibility of tooth position at each time of color measurement, a mold was made for each specimen from additional silicone (mega PINKSIL N2; megadental GmbH, Büdingen, Germany), and a custom-fabricated measuring station was fabricated carrying the spectrophotometer device at an accurate reproducible position with the specimen’s silicone mold, with the probe tip of the device just touching the middle part of the buccal surface of the crown of the tooth. The spectrophotometer was calibrated according to the manufacturer’s recommendations before the color measurement of each group, and all the measurements were performed by one single operator.

Color measurements were taken for each specimen at the middle of the buccal surface of the crown in a dark room at six intervals: T0 (before material placement), T1 (after 24 h of material placement), T7 (after 1 week), T30 (after 1 month), T90 (after 3 months), and T180 (after 6 months). The excess water from the crown surface was removed briefly by air-drying for 1 second and each specimen was measured twice, and the mean value was calculated at each measurement time for each specimen.

The mean values of L*, a*, and b* were taken and the color change (∆E*) of each specimen was calculated for each interval using the following equation [20]:

$$\Delta {\text{E}} = \left[ {\left( {\Delta {\text{L}}*} \right)^{{\text{2}}} + \left( {\Delta {\text{a}}*} \right)^{{\text{2}}} + \left( {\Delta {\text{b}}*} \right)^{{\text{2}}} } \right]^{\raise.5ex\hbox{$\scriptstyle 1$}\kern-.1em/ \kern-.15em\lower.25ex\hbox{$\scriptstyle 2$} }$$

L* parameter indicates lightness from 0 (black) to 100 (white); a* represent red (+ 80a*) to green (− 80a*); and b* represent yellow (+ 80b*) to blue (− 80b*) [16]. Color change (ΔE) values beyond 3.7 were considered clinically perceptible [21].

ΔE after material placement was calculated first to see the effect of transmission of the materials through tooth structure. Then ΔE was calculated for the following intervals: after 1 week, 1 month, 3 months, and after 6 months. The parameter measurements at T1 (after 24 h of experimental materials placement) were considered the baseline.

### Statistical analysis

Mixed model—repeated measure ANOVA was used to evaluate the effect of the study variables (materials, and time) on color change (∆E). Akaike information criterion (AIC) was used to determine the model fit. Post hoc analysis was performed by Bonferroni’s test for pairwise comparison with a significant level of 0.05.

## Results

### Tooth color assessment by ∆Ε

Table 1 presents ∆Ε values of the tooth samples after placement of materials T1 (with saline or blood) relative to T0 before material placement. Placement of the materials into the pulp chamber had changed the color of the teeth whether with saline or with blood. In the saline groups this was obvious for the GMTA group in particular (3.97) followed by the WMTA (2.96), and the change for the other groups was much less. While for the blood groups, the WMTA group was the most affected reaching a value of 5.16 which is even greater than the control (4.68) that had only a cotton pellet moistened with blood in the cavity of the pulp chamber. The second that was affected by the color change was TotalFill but with a relatively large standard deviation (3.46 ± 2.11), and the least color change was for Biodentine, but again with a relatively large standard deviation (1.98 ± 1.29). Therefore, ∆Ε was calculated for the different time intervals from T1 as base line which represents the tooth color after 24h of material placement.

**Table 1:** Mean ± SD for ∆Ε values after 24 h of material placement for experimental material groups tested with saline or with blood.

	Tested materials groups					
	Control*	GMTA*	WMTA*	TotalFill*	Biodentine	TheraCal
Saline	1.98 ± 0.83	3.97 ± 1.22	2.96 ± 1.13	1.95 ± 1.24	1.45 ± 0.42	1.92 ± 0.99
Blood	4.68 ± 1.93	2.83 ± 0.82	5.16 ± 2.62	3.46 ± 2.11	1.98 ± 1.29	2.40 ± 1.71

*Groups in which the ∆Ε values for saline were significantly different from the ∆Ε values for blood (*p* < 0.05).

#### ∆*Ε* for Saline groups

**Fig. 2:**
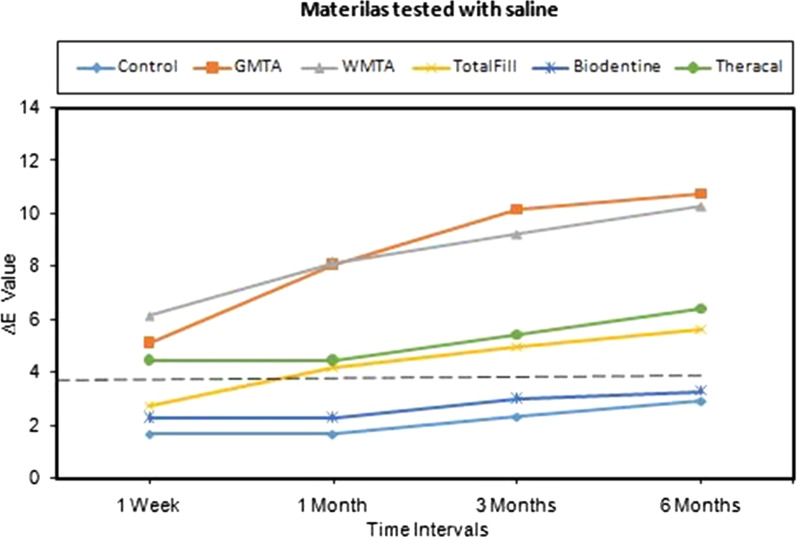
Mean values of ∆Ε for tested materials with saline at various time intervals. Interrupted line (----) represent the clinically perceptible level. Control; saline only

The mean values of ∆Ε for the groups that were tested with saline at the four intervals including the negative control group are illustrated graphically in Fig. 2. Overall, Biodentine had caused the least color change, and the GMTA and WMTA had caused the highest color change, while the TheraCal and TotalFill had fallen in between but more toward the Biodentine and the control. All the materials had mean values of ∆Ε above the clinically perceptible level (3.7) except Biodentine was consistently below that level.

Mixed model—repeated measures ANOVA for ∆E showed that; materials, time, and the interaction between them had a highly significant effect (*p* < 0.001). Bonferroni multiple comparison test was then performed to compare estimated marginal means of the ∆E for different tested materials irrespective of the time intervals. The comparison showed no significant differences between: Control with Biodentine (*p* = 1.00); GMTA with WMTA (*p* = 1.00); and between TotalFill with TheraCal (*p* = 0.334), while the other comparisons between the material groups were found to be significant (Table 2A). Furthermore, comparisons were also performed to compare the estimated marginal means of the ∆E for different time intervals irrespective for tested materials, which revealed significant differences between all the time intervals except the difference between 3 months with 6 months that was not significant (*p* = 0.217) (Table 2B).

**Table 2:**
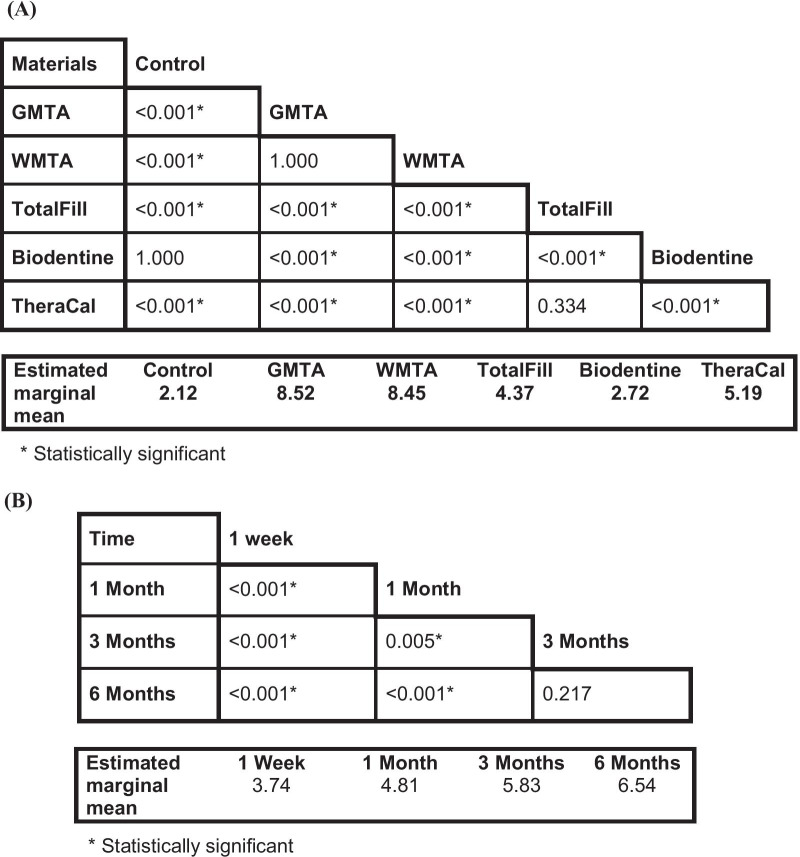
P values of the pairwise comparison / Bonferroni test for the estimated marginal mean of ∆Ε values; (A) between the groups of materials tested irrespective of time. (B) between different time intervals irrespective of materials, Saline Groups

#### ∆*Ε* for blood groups

**Fig. 3:**
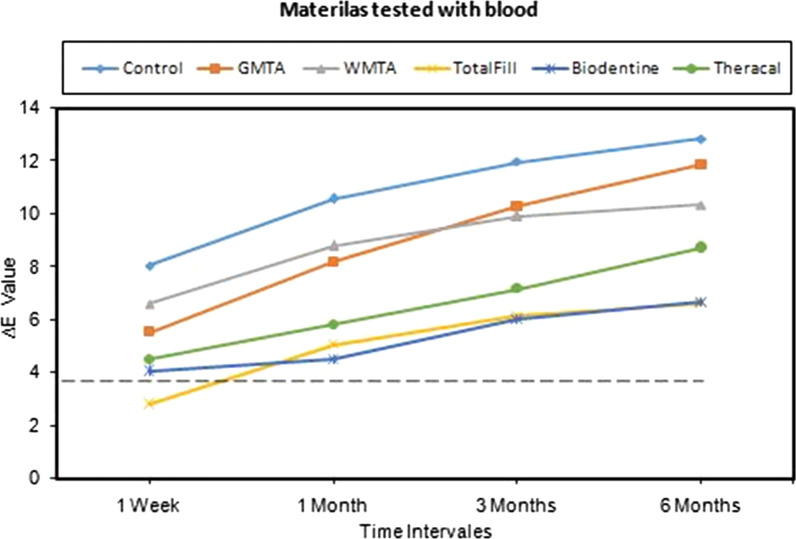
Mean values of ∆Ε for tested materials with blood at various time intervals. Interrupted line (----) represent the clinically perceptible level. Control; blood only

The mean values of the ∆Ε for the groups that were tested with blood including the positive control at the four intervals are illustrated graphically in Fig. 3. Overall, the GMTA and WMTA had caused the highest color change, and the Biodentine and TotalFill were found to cause the least color change, while TheraCal had fallen in between. Interestingly all the ∆Ε values were above 3.7 (clinically perceptible threshold) except for TotalFill at one week (2.82) but this could be because the value of ∆Ε of the TotalFill at the base line T1 was high.

Mixed model—repeated measures ANOVA for the values of the ∆E showed that materials and time both had a highly significant effect (*p* < 0.001), but the interaction between them was not statistically significant (*p* = 0.42). Bonferroni multiple comparison test showed no significant difference between; GMTA with WMTA (*p* = 1.00), TotalFill with Biodentine (*p* = 1.00), and between Biodentine with TheraCal (*p* = 0.105), while other comparisons were statistically significant (Table 3A). Furthermore, the estimated marginal means of the ∆E comparison for different time intervals irrespective for tested materials, showed again significant differences between all the time intervals except the difference between 3 months with 6 months that was not statistically significant (*p* = 0.184) (Table 3B).

**Table 3:**
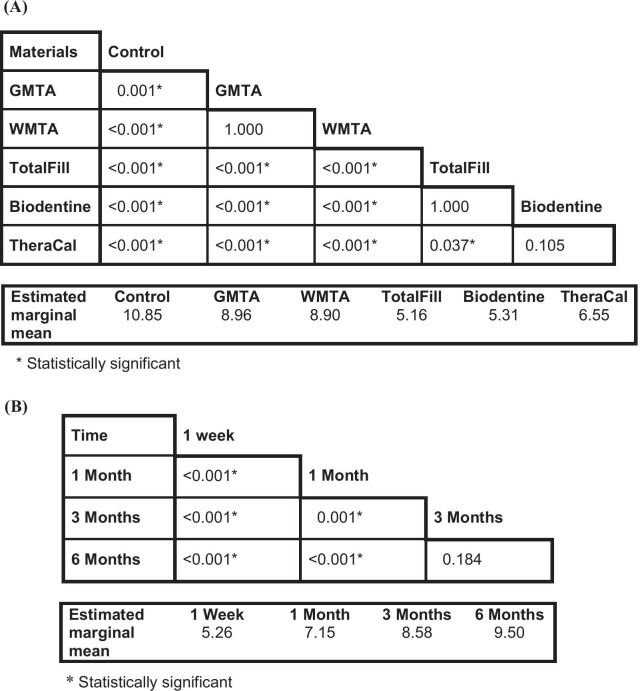
P values of the pairwise comparison / Bonferroni test for the estimated marginal mean of ∆Ε values; (A) between the groups of materials tested irrespective of time. (B) between different time intervals irrespective of materials, Blood Groups

### Effect of blood on color change by materials

**Fig. 4:**
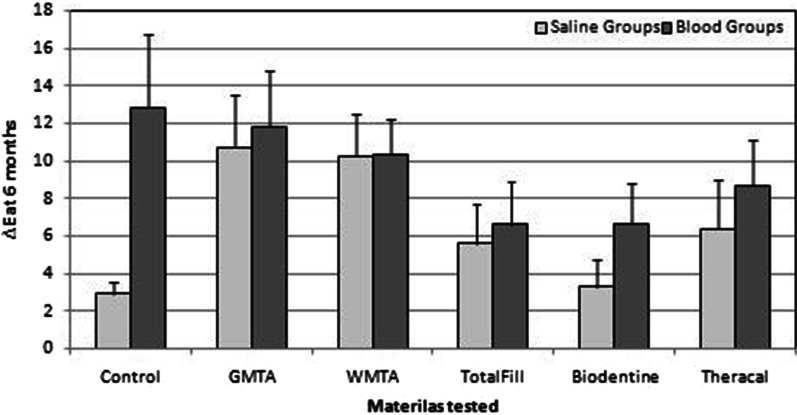
Mean values of ∆Ε for the tested materials of the saline groups and blood groups at the 6 months time, error bar represent the SD

Further analysis was carried out to determine the influence of blood contamination on the discoloration effect of the materials at the 6 month time (saline groups vs. blood groups). It was found that the presence of the blood increased overall the color change of the teeth, but not to the same degree for all materials as seen in Fig. 4. Biodentine was the most to be affected (*p* < 0.001) followed by Theracal (*p* = 0.03), while WMTA was the least to be affected (*p* = 0.95) followed by GMTA (*p* = 0.35) then TotalFill (*p* = 0.27).

## Discussion

The results of this study indicate that the tested materials discolored tooth structure to various degrees, which was observed by the variation in the ∆Ε values in relation to the different material compositions. GMTA Angelus was intended as the positive control group for the materials tested, however, the difference between ProRoot WMTA and GMTA Angelus was not significant, having the most severe pattern in color change that was caused by both. Although the GMTA formula was modified by reducing the metal oxides such as iron, aluminum, and magnesium oxide which may take part in color change [22], the resulting formula which is the WMTA, is reported to cause same discoloration effect as the GMTA [8, 23], and as it was found in the present study.

The discoloration induced by MTA has been reported to be caused by bismuth oxide which is added as a radiopacifier. One of the possible mechanism of discoloration caused by bismuth oxide, is that oxidation of bismuth oxide destabilizes the oxygen in its formulation, and reacts with carbon dioxide to produce bismuth carbonate which is light sensitive that results in a black precipitate when exposed to light [6]. Another theory is that bismuth oxide interacted with collagen and converted into a black precipitate [5].

In the other materials tested, the bismuth oxide was replaced by other constituents to serve as radiopacifier such as; zirconium oxide in Biodentine, tantalum and zirconium oxide in TotalFill, and barium zirconate in TheraCal. Thus, the discoloration in these three material groups was significantly less than WMTA and GMTA whether the materials were tested with saline or with blood. Furthermore, Biodentine in the saline group hardly caused any discoloration almost like the negative control, while the TotalFill and TheraCal have caused significantly more, but when the materials were tested with blood the difference between these three groups were not statistically significant but still significantly less than the MTA groups. These results agree with previous studies where the most color change was related to the MTA materials more than Biodentine or premixed bioceramic (EndoSequence RRM) [24–27]. Akbulut et al. (1917), reported that blood is the main causative factor in tooth color change rather than the type of calcium silicate cement used, they found no significant difference in tooth discoloration induce by MTA or Biodentine, but after bleaching, the teeth in the Biodentine group were found to be significantly more whitened than those teeth in the MTA groups [28].

The Biodentine groups also showed color stability over time particularly in the saline group. The color stability of  Biodentine has been reported before [6, 29], and discoloration of the teeth with darkening of the coronal structure after one year was more evident for the MTA samples than with the Biodentine [30]. In another study using bovine teeth, Marciano et al. [5] found that teeth filled with Portland cement that have different radiopacifiers other than bismuth oxide (zirconium oxide and calcium tungstate) have exhibited color stability, and no color change have occurred after zirconium oxide and calcium tungstate were placed in contact with collagen, but, discoloration was observed when the authors placed bismuth oxide in contact with the collagen [31]. Similar findings have been reported more recently demonstrating again that, calcium silicate cement containing bismuth oxide was found to cause the highest color change compared to those with zirconium oxide or calcium tungstate as radiopacifiers when the cements were placed into extracted human premolars in contact with blood [32].

The discoloration caused by TheraCal and TotalFill was more than that caused by Biodentine in the saline test groups but when blood was added, the Biodentine became very similar to the TotalFill. The reason for this difference in discoloration potential is due to the differences in chemical structure of these three materials, some of which are affected by the blood more than the others. All three materials have zirconium compounds in their composition to act as radiopacifier instead of bismuth oxide, but Biodentine has some elements not present in others which include calcium carbonate, calcium oxide and iron oxide. On the other hand TotalFill differs in the presence of calcium phosphate in its composition, and other elements that are present only in the composition of TheraCal are barium sulfate, fumed silica, Sr glass, and resin. Further evaluations are required to investigate the chemical reactions involved in the color change of these materials.

This study revealed that after contamination of the specimens with blood, overall the estimated marginal means of ∆Ε were increased for the materials particularly; for the Biodentine (2.59) and TheraCal (1.36) and to a lesser extent for the TotalFill (0.79), but very little for the MTA groups (G: 0.44, W: 0.45). Apart from the hemostasis whether if it is fully archived or not, these materials come in contact with vital tissue, in vital pulp therapy procedures or in revascularization when applicable. Some of the components of these materials may have different reactions with blood and vital tissue. Thus, clinical studies should be performed investigating the influence of bleeding time of pulpal tissue and the thickness of remaining coronal tooth structure in tooth discoloration.

In the present study, comparing the ∆E values at 6 months for the experimental materials in both the saline and the blood groups, it could be noticed that the ∆E at 6 months for the groups of materials containing bismuth oxide (GMTA and WMTA) has not increased in greater amounts in the presence of blood relative to the other materials. This could be related to the high discoloration potential and high ∆E values caused by the material itself, irrespective to the presence of blood. While in the other groups of; TotalFill, Biodentine, and TheraCal, where the bismuth oxide were replaced with other radiopacifiers, the increase in the discoloration (∆E at 6 months) when tested with blood was almost double for Biodentine, lesser increase for the TheraCal and the least increase was for TotalFill. This could be related to the fact that both TotalFill and TheraCal are premixed  materials with greater homogeneity compared to Biodentine which comes in Powder-liquid form that should be mixed in an amalgamator. This could make the mixture easy to entrap the blood molecules that could penetrate easily within the particles of the material, while this could be less possible in the premixed materials.

Previous studies reported that the porosities in the microstructure of calcium silicate-based materials may uptake blood components and cause discoloration [33]. Also erythrocyte penetration into the tooth structure could intensify the discoloration as well [34]. This was obvious in the positive control group where the pulp chamber cavity was filled with a cotton pellet moistened with 12 μL blood, which showed a very high value of color change (∆E) at 6 months (Fig. 4). It has been reported that sealing the dentinal tubules of the pulp chamber of bovine incisors with a dentin bonding agent, in a simulating regenerative endodontic treatment with the use of antibiotic and blood clot or platelet-rich fibrin, color change of coronal tooth structures filled with MTA or Biodentine was not different at 6 month follow-up [35].

Therefore, it is better for these materials to be used only after achieving complete hemostasis to decrease the potential of discoloration effect. Furthermore the presence of calcium carbonate in the Biodentine may also make the material more affected by the blood than the other materials tested. This needs more investigation as stated before. Indeed, Palma et al. [29] found that after 6 months, MTA with saline had higher ∆E value by 5.08 than samples of Biodentine with saline, while the MTA with blood had 3.65 higher ∆E value than the Biodentine with blood, thus the blood had more effect on the Biodentine samples than on the MTA samples as reported in their results.

It could be noticed that overall in both groups (saline and blood) there were no significant differences between the 3 months and 6 months times, thus, the discoloration effect of materials becomes less after 3 months, although this was not that clear for the TheraCal in particular as seen in Figs. 2 and 3.

Overall, and based on the result of this study, the calcium silicate-based materials that were tested in this study have caused tooth discoloration with large differences between them; those with bismuth oxide in their composition (GMTA, and WMTA) have caused the most severe discoloration. Therefore, MTA with bismuth oxides should be avoided in the anterior teeth. Contact with blood has increased the discoloration caused by the materials overall, therefore, in pulp capping procedures, hemostasis should be achieved to reduce the discoloration potential of the calcium silicate materials, even if they do not have bismuth oxide in their compositions.

## Conclusions

Under the conditions of this in vitro study, the following could be concluded:

- Calcium silicate-based materials with bismuth oxide in their composition (GMTA, and WMTA) have caused the most severe discoloration, while Biodentine was found to cause the least. The effect of TotalFill and TheraCal was moderate overall.
- Blood contact has increased the discoloration effect of all the materials tested
- All the discolorations caused by Biodentine in the absence of blood were below the clinically perceptible level.
- In contact with blood, Biodentine was the most to be affected, while TotalFill was the least, and both had almost the same value of ∆E at the 3 and 6 months time.

## Data Availability

The samples with materials are stored at the main author laboratory, and all the materials and the raw data are available upon request from the corresponding author, due to the large number of the raw data.

## Abbreviations

MTA: Mineral trioxide aggregate
GMTA: Gray MTA
WMTA: White MTA
